# An International Review of Motor Vehicle Collision Risk with Medical Conditions in Older Adults

**DOI:** 10.1093/geroni/igab046.3176

**Published:** 2021-12-17

**Authors:** Mark Rapoport, Joanne Wood, Jamie Dow, Desmond O'Neill, Judith Charlton, Sjannie Koppel

**Affiliations:** 1 University of Toronto, Toronto, Ontario, Canada; 2 Queensland University of Technology, Brisbane City, Queensland, Australia; 3 SAAQ, Quebec City, Quebec, Canada; 4 Trinity College Dublin, Dublin, Dublin, Ireland; 5 Monash University, Clayton, Victoria, Australia

## Abstract

The objective was to examine the impact of seven categories of medical illness on risk of Motor Vehicle Collisions (MVC) in older adults. In late 2019, a systematic review of the MVC risk associated with alcohol use disorders, psychiatric disorders, epilepsy, diabetes, hearing loss, vision disorders and sleep disorders was conducted. A total of 64,720 titles were screened, and 138 articles were included. Of these, only thirteen pertained to older adults, only six showed increased MVC risk in at least one condition, and only seven were rated of “Good” quality. Hearing impairment was associated with MVC only if associated with visual acuity or contrast sensitivity impairments (RR 1.52, 95% CI 1.01-2.3 and RR 2.41, 95% CI 1.62-3.57, respectively). A high depression score was associated with increased MVC (RR 1.5, 95% CI 1.1-2.1) in one study, but a similar relationship was not found in two other studies. Glaucoma increased at-fault MVC risk (RR 1.65, 95% CI 1.20-2.28) in one study, but no relationship was found in another. Visual field loss increased MVC risk in three of four studies (RR or HR ranging from 1.31 to 2.32). One negative study each were identified for alcohol use disorders, age-related macular degeneration, any eye disease, or any psychiatric disorder, and four negative studies were identified for reduced visual acuity. No studies of older adults were found for epilepsy or sleep disorders. Interpretation of MVC risk in older drivers with medical illness is rendered challenging by the paucity and quality of studies.

